# A rare case of tropical chronic pancreatitis with giant pseudocyst: Case report

**DOI:** 10.1016/j.amsu.2021.102947

**Published:** 2021-10-14

**Authors:** Irwan, M. Iqbal Rivai, Juni Mitra, Avit Suchitra, Rini Suswita, Aulia Janer, Edo B. Tantyo

**Affiliations:** a Division of Digestive Surgery, Department of Surgery, Faculty of Medicine Andalas University - M.Djamil General Hospital, West Sumatera, 25171, Indonesia; b General Surgery Resident, Faculty of Medicine, Andalas University – M. Djamil General Hospital, West Sumatera, 25171, Indonesia

**Keywords:** Tropical chronic pancreatitis (TCP), Pseudocyst pancreas, Partington-rochelle procedure, Case report

## Abstract

**Background/objective:**

Tropical chronic pancreatitis (TCP) is common in developing countries and is defined as a juvenile form of chronic calcific non-alcoholic pancreatitis. Pseudocysts occur in 20–40% of chronic pancreatitis. TCP with pseudocyst has not been reported yet, so we represent this rare case to broaden the horizons regarding pancreatitis.

**Case presentation:**

A 16-year-old woman suffered a painful lump in the upper abdomen. She came from a low-income family and frequently consumed cassava. There was intolerance of glucose in which admission blood sugar level of the patient increased by 179 mg/dl. An abdominal CT scan showed a mass around the pancreas, 20 cm in diameter, and located in retro-gastric. There were multiple ductal calculi along the major pancreatic duct with the largest stone was 3 cm in the pancreatic head. Longitudinal pancreaticojejunostomy (Partington-Rochelle procedure) has been performed and histopathological results appropriate with a pancreatic pseudocyst.

**Clinical discussion:**

TCP with a giant pseudocyst is an interesting case report that has not been reported yet. This case met the clinical characteristics of TCP, such as young women, malnourished, history of cassava consumption, abdominal pain, and intolerance of glucose. A surgical intervention provides a satisfactory result to the patient.

**Conclusion:**

Tropical chronic pancreatitis is a rare case. A pseudocyst adds the uniqueness of this case that has never been reported before. Appropriate management can provide satisfactory results and improve the quality of life for patients.

## Highlights

• Tropical chronic pancreatitis is chronic calcific non-alcoholic pancreatitis.
• Tropical chronic pancreatitis (TCP) is common in developing countries.
• Tropical chronic pancreatitis accompanied by giant pseudocysts is a rare case.
• Partington-Rochelle procedure as surgical intervention for TCP.

## 1 Introduction

Tropical chronic pancreatitis (TCP) is common in developing countries. TCP is defined as a juvenile form of chronic calcific non-alcoholic pancreatitis [1,2]. In 1950, Zuidema first reported cases of TCP in young Indonesians [3,4]. The largest incidence of TCP has been reported in South India, in addition, 55% of TCP patients also have diabetes [5]. The etiology is still unclear, the current consensus says there are genetic factors and cassavas consumption. TCP is included in the TIGAR-O classification of chronic pancreatitis as idiopathic etiology [6]. It is associated with the development of insulin-dependent diabetes mellitus in people under 30 years [2,7].

A pancreatic pseudocyst is a localized fluid collection surrounded by a well-defined wall of fibrous tissue [7]. In incidence, 20–40% of pseudocysts occur in chronic pancreatitis, 70–78% in alcohol-induced chronic pancreatitis, and 6–16% in idiopathic chronic pancreatitis [8]. We did not find any previously reported cases of TCP with pseudocysts. Therefore we represent this rare case to broaden the horizons regarding pancreatitis. This work has been reported in line with The SCARE 2020 criteria [9].

## 2 Case Presentation

A 16-year-old woman, a student in a rural area, suffered intractably pain in the upper abdomen for three weeks. Pain radiated to the back, progressive and persistent. The pain was felt four months before admission, but the patient ignored it and tried to self-medicate with over-the-counter drugs. There was a lump in the upper abdomen for two months, steatorrhea, frequent nausea, and vomiting. The patient comes from a low-income family, often consumes cassava, and has no history of jaundice, alcohol consumption, and no history of abdominal trauma.

**Fig. 1:**
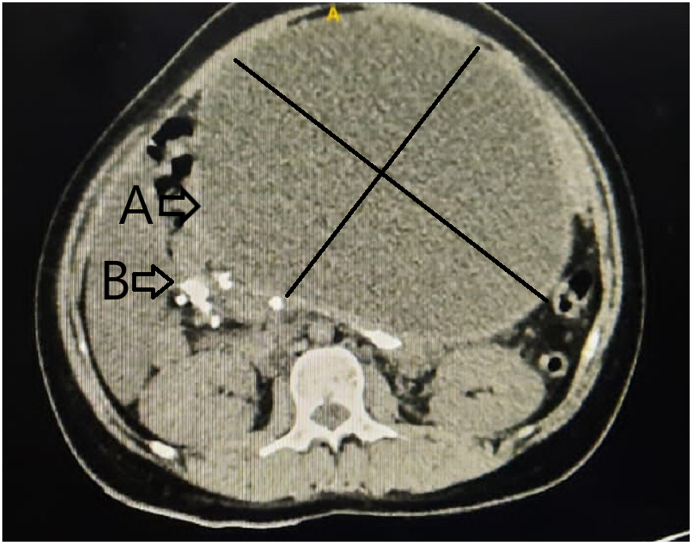
Abdominal CT scan demonstrated: (A) Large pseudocyst pancreas (B) Multiple stones in the major pancreatic duct and the largest stone in the pancreatic head.

Vital signs were stable, low BMI (17,6 kg/m2), without jaundice. A lump in the epigastric region with a cystic consistency, smooth, fixed, and tenderness mass. The blood sugar level increased by 179 mg/dl. Abdominal CT scan showed a mass around the pancreas, 20 cm in diameter, and located in retro-gastric. There were multiple ductal calculi along the major pancreatic duct, and a large stone was 3 cm in diameter in the pancreatic head. There was no suppression of the common bile duct, and the liver was within normal appearance (Fig. 1).

**Fig. 2:**
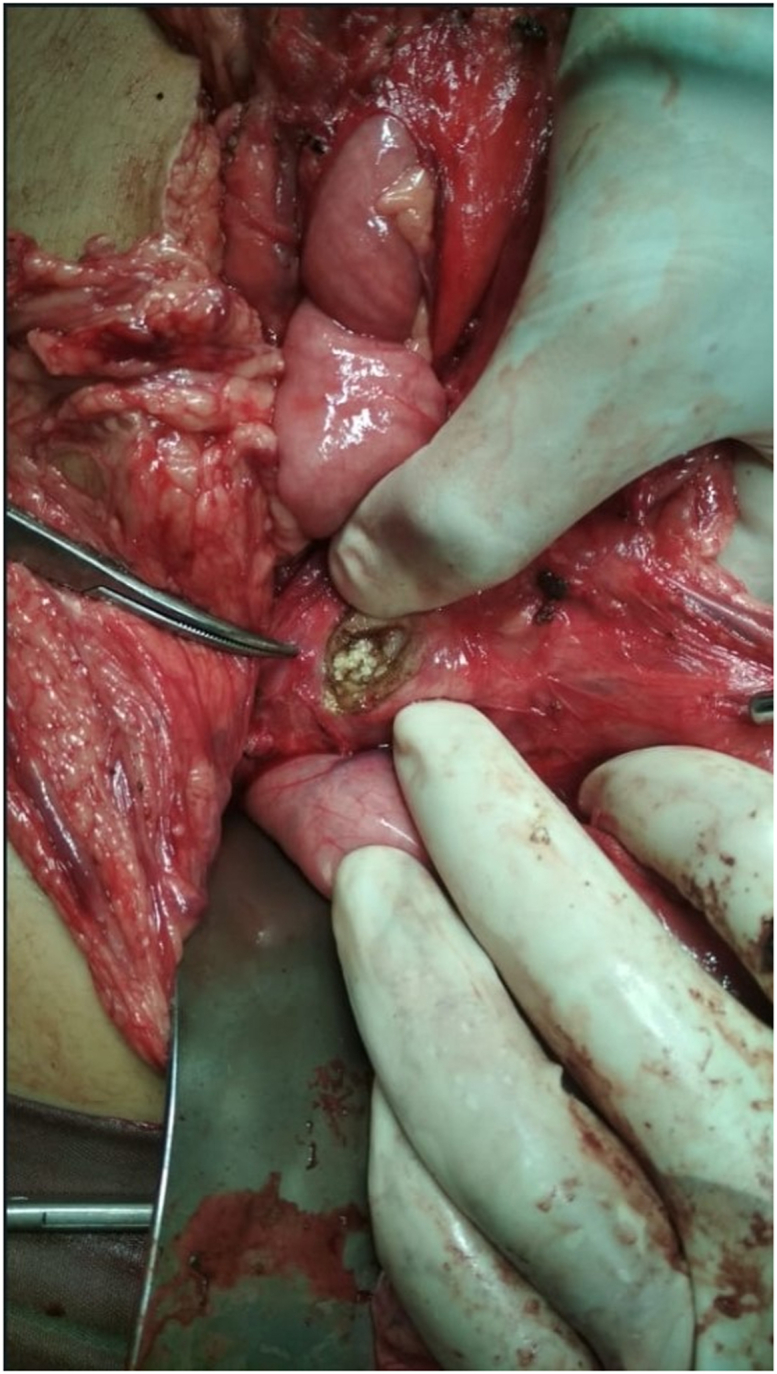
The largest stone in the pancreatic head.

**Fig. 3:**
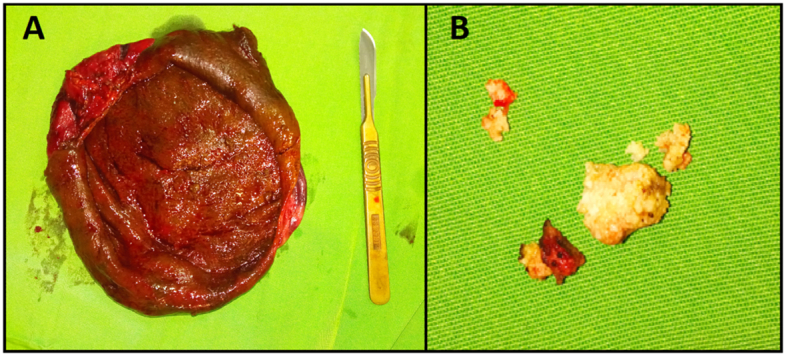
A. Giant pseudocyst after resection B. Multiple stones.

We performed the Partington-Rochelle procedure with excision of the pseudocyst pancreas. Briefly, after midline incision, there was an encapsulated mass in the retro-gastric. the gastrocolic ligament is dissected and divided for the exposure of the entire anterior surface of the pancreas and to trace the pancreatic duct. The cystic mass diameter was 20 cm and not connected to the major pancreatic duct. Palpable multiple stones along the major pancreatic duct. We decided to excise the cystic mass instead of only drainage because the cyst was not independently diagnosed. Make a long longitudinal opening of the duct to ensure full decompression. After the removal of ductal calculi and concretions, resected or cored out pancreatic parenchyma should be sent for frozen section to rule out occult malignancy. There was a dilatation along the pancreatic duct with a diameter of more than 7 mm, but in the pancreatic head, an impacted stone and ductal stenosis were found (Fig. 2, Fig. 3).

Next, a 50 cm Roux-en-Y loop is constructed and the jejunum is divided 20–30 cm distal to the ligament of Treitz. Subsequently, a side-to-side jejunojejunostomy is performed between the proximal jejunum and the distal part of the Roux. Then the proximal part of the Roux-en-Y is passed through retro-colic and positioned next to the lateral opening of the pancreatic duct. An enterotomy is made along the antimesenteric border of the jejunal loop, slightly shorter than the length of the duct incision as the intestine will stretch. We performed the pancreaticojejunal anastomosis with a single layer of a running 4–0 absorbable suture between the full-thickness small bowel and pancreatic duct. Histopathological results were a cyst wall without epithelial lining, consisting of a connective tissue stroma containing infiltrated lymphocytes, PMN leukocytes, and histiocytes. These results were appropriate with a pancreatic pseudocyst. The length of stay was seven days with the uneventful condition. In one month follow-up, abdominal pain was absent, and the patient's blood sugar levels were within normal limits.

## 3 Discussion

TCP is different from alcoholic pancreatitis (ACP). The gender percentage of patients with TCP is 70:30 for males and females, socioeconomic status is poor, and diabetes status is accelerated. The presence of diabetes and pancreatic stones is more than 90%, and in TCP, also found ductal dilatation [10]. The criteria of TCP are; age <30 years, presence of malnutrition BMI <18 kg/m2, presence of glucose intolerance or diabetes, and no other etiology found. Several factors have been suggested as etiology, such as malnutrition, cassava consumption (cyanogen toxicity), genetic factors, oxidative stress, free radical injury, and trace element deficiency. The classical triad of clinical presentation in TCP: abdominal pain, maldigestion leading to steatorrhea, and diabetes [2,3].

In this case report, a 16-year-old girl with abdominal pain, glucose intolerance, and improper growth or malnutrition. The patient had glucose intolerance with a random blood sugar entry of 179 mg/dl. The natural history of TCP is initially characterized by glucose intolerance and eventually fibrocalculus pancreatic diabetes (FCPD) [1]. She came from a low-income family and frequently consumed cassava. The association of malnutrition and consumption of cassava was confirmed in TCP. Cassava root contains 65 mg of toxic glycosides/l00g such as linamarin and lotaustralin, which are alleged to cause pancreatic injury [11]. Cyanide must be metabolized to thiocyanate in the liver by sulfur transferases (mainly rhodanese) and excreted in the urine [12]. In patients who are malnourished, sulfur-containing amino acids such as methionine and cystine are deficient, so when these patients consume cassava, they are theoretically susceptible to chronic pancreatitis leading to TCP [1]. Several studies suggest consuming cassava as the etiology of TCP, but it is still unclear as a single etiology. Mcmillan and Geevarghese reported that mice developed transient hyperglycemia after the administration of cyanide [13]. Contrary, Mathangi et al. found no pancreatitis or diabetes in mice which was fed cassava even after one year of consumption [14].

In more than 90% of TCP patients, pancreatic calculi may be detected mainly in the later stages, stones vary small to large (20gr), smooth, rounded, or stag-horn-like [1,11]. In this case, the largest stone was 3 cm in the pancreatic head and also found dilatation of the major pancreatic duct. The calculus in alcohol and hereditary pancreatitis is similar, well-shaped, mottled, and poorly defined. While the TCP stone is solid and has clear boundaries. Dilatation of the pancreatic duct is less common in alcoholic and hereditary pancreatitis than in TCP [15].

This case is unique because pseudocysts accompanied the TCP. The majority of pancreatic pseudocyst occurs as a common complication of chronic pancreatitis, but they may occur during acute pancreatitis or pancreatic trauma, or following pancreatic surgery. Pancreatic pseudocyst does not lead to malignancies [16]. There are numerous classification systems for pseudocysts. Recently, Pan et al. proposed a new classification based on the anatomical location and clinical manifestation of the pseudocysts, along with the relationship between the cyst and the pancreatic duct [16]. There are no current guidelines for the treatment of pancreatic pseudocysts [17]. We did not find any previously reported cases of TCP with pseudocysts. Nevertheless, Yuqing Gu reported pseudocysts in the ACP [18]. The Partington-Rochelle procedure was performed by longitudinal incision along the major pancreatic duct and side-to-side pancreaticojejunostomy [19,20]. The strongest reason for choosing the Partington-Rochelle procedure was the need for a longitudinal incision in the pancreas for evacuation of multiple stones along the major pancreatic duct. A randomized controlled trial showed this procedure has a better quality of life and pain relief [21]. On our follow-up, we found no symptoms of abdominal pain in the patient, and the random blood glucose was normal.

## 4 Conclusion

Tropical chronic pancreatitis is a rare case. A pseudocyst adds the uniqueness of this case that has never been reported before. Appropriate management can provide satisfactory results and improve the quality of life for patients.

## Provenance and peer review

Not commissioned, externally peer-reviewed.

## Ethical approval

Ethical approval has been exempted by our establishment.

## Sources of funding

None.

## Author contribution

Irwan: operator in surgery, data collection, writing the paper. M. Iqbal Rivai: operator in surgery, support in clinical dan surgical analysis. Juni Mitra, Avit Suchitra, and Rini Suswita: support in clinical dan surgical analysis. Aulia Janer: data collection and writing the paper. Edo B Tantyo: data collection.

## Consent

Written informed consent for publication of their clinical details and clinical images obtained from the patient.

## Research registration

None.

## Guarantor

Irwan, Aulia Janer.

## Declaration of conflict interest

None.
